# Hypoxia-induced HIF-1α promotes *Listeria monocytogenes* invasion into tilapia

**DOI:** 10.1128/spectrum.01405-23

**Published:** 2023-09-08

**Authors:** Jing Wang, Zhan He, Mingzhu Cui, Jing Sun, Lingli Jiang, Nanxi Zhuang, Fuxin Zhu, Xian Zhang, Houhui Song, Changyong Cheng

**Affiliations:** 1 Key Laboratory of Applied Technology on Green-Eco-Healthy Animal Husbandry of Zhejiang Province, Zhejiang Provincial Engineering Laboratory for Animal Health Inspection & Internet Technology, Zhejiang International Science and Technology Cooperation Base for Veterinary Medicine and Health Management, China-Australia Joint Laboratory for Animal Health Big Data Analytics, College of Animal Science and Technology & College of Veterinary Medicine of Zhejiang A&F University, Hangzhou, Zhejiang, China; 2 Ningbo College of Health Sciences, Ningbo, Zhejiang Province, China; Institute of Hydrobiology, CAS, Wuhan, China

**Keywords:** *Listeria monocytogenes*, hypoxic environments, invasion, tilapia

## Abstract

*HIF-1α* is a nuclear transcription factor, and its activity is tightly regulated by the level of available oxygen in cells. Here, we investigated the roles of *HIF-1α* in the invasion of *Listeria monocytogenes* into tilapia under hypoxic environments. We found that the expression levels of *HIF-1α* in examined tissues of hypoxic tilapia were significantly upregulated, indicating that the tissue cells have been in hypoxic conditions. After 24-h infection with *L. monocytogenes*, we found that bacterial burden counts increased significantly in all examined tissues of hypoxic fish. To explore why the bacterial count increased significantly in the tissues of hypoxic fish, we modulated *HIF-1α* expression through RNAi technology. The results indicated that *c-Met* expression levels were positively related to *HIF-1α* expression. Since c-Met is the receptor of InlB that plays critical roles in the adhesion and invasion of *L. monocytogenes*, the ∆*InlB* strain was used to further explore the reason for the significant increase in bacterial counts in hypoxic fish. As expected, the decrease in the adhesion ability of ∆*InlB* suggested that InlB mediates *L. monocytogenes* infection in tilapia. After being infected with ∆*InlB* strain, we found that the bacterial counts in hypoxic fish were not affected by hypoxic conditions or *HIF-1α* expression levels. These findings indicate that *HIF-1α* may promote the internalization of InlB by upregulating *c-Met* expression and therefore contributes to the invasion of *L. monocytogenes* into hypoxic tilapia.

**IMPORTANCE:**

*Listeria monocytogenes* is a zoonotic food-borne bacterial pathogen with a solid pathogenicity for humans. After ingestion of highly contaminated food, *L. monocytogenes* is able to cross the intestine invading phagocytic and nonphagocytic cells and causes listeriosis. China is the world’s largest supplier of tilapia. The contamination rate of *L. monocytogenes* to tilapia products was as high as 2.81%, causing a severe threat to public health. This study revealed the underlying regulatory mechanisms of *HIF-1α* in the invasion of *L. monocytogenes* into tilapia under hypoxic environments. This study will be helpful for better understanding the molecular mechanisms of hypoxic environments in *L. monocytogenes* infection to tilapia. More importantly, our data will provide novel insights into the prevention and control of this pathogen in aquaculture.

## INTRODUCTION

China is the world’s largest supplier of tilapia (1). With the increasing tilapia farming scale and the increasing intensification level, low dissolved oxygen content often occurs in farming ponds. To cope with an anoxic environment, biological bodies have formed a series of regulatory mechanisms, among which hypoxia-inducible factor-1 (HIF-1), as a nuclear transcription factor produced by hypoxia-induced cells, is a master regulator of oxygen sensitivity (2, 3). Under normoxic conditions, the activity of the HIF-1 alpha subunit (*HIF-1α*) is governed by a repression mechanism (4). *HIF-1α* is activated when organisms are in low-oxygen environments and work together with the beta subunit to directly regulate the transcription of specific genes involved in responses to hypoxia. Respiratory, metabolic, and immune responses may be affected when *HIF-1α* is activated (5 – 7).

In 2017, a survey found that 2.81% of tilapia exports were contaminated with *Listeria monocytogenes*, which is far higher than the amount of contamination at *Salmonella* (0.20%), *Vibrio parahaemolyticus* (0.80%), and *Vibrio cholerae* (0.51%), which seriously threatened human public health security (8). *L. monocytogenes* is a zoonotic food-borne bacterial pathogen with a solid pathogenicity for humans (9). After ingestion of highly contaminated food, *L. monocytogenes* is able to cross the intestine invading phagocytic and non-phagocytic cells and causes listeriosis (10). *L. monocytogenes* is a typical intracellular pathogen, and the primary prerequisite for its pathogenicity to the host is that it can successfully invade the host cell. Internalin A (InlA) and InlB, as the critical virulence factors, play vital roles in the process of adhesion and invasion of *L. monocytogenes* (11). InlA is a unique surface protein of *L. monocytogenes*, of which the primary function is to mediate bacterial invasion into host cells and internalize into phagosomes (12). This process is the prerequisite for establishing infection between *L. monocytogenes* and the host. InlA mediates bacterial entry into host cells by specifically binding to E-cadherin (13 – 15). E-cadherin is a specific receptor on the surface of host cells, essential for maintaining tight and interstitium junctions (16). In the hypoxic regions of breast cancer, cervical cancer, gastric cancer, and other tumor cells, the abnormal expression of E-cadherin can lead to the abnormal regulation of the related signaling pathway, which will affect the adhesion, invasion, metastasis, and survival of cells, and the abnormal expression of E-cadherin is often closely related to the overexpression of *HIF-1α* in the hypoxic environment of tumors (17, 18). In the internalization of *L. monocytogenes*, InlB also plays an irreplaceable role. Similar to InlA, InlB can specifically bind to cell surface hepatocyte growth factor receptor (c-Met) to mediate the invasion of *L. monocytogenes* into most host cells (19, 20). Cell surface c-Met is a specific receptor for InlB and one of the downstream genes in the *HIF-1α* signaling pathway. A study of liver cancer cells showed that the hypoxia environment in tumors could promote the upregulation of the c-Met transcription level, and the upregulation process is regulated by *HIF-1α*, and the expression level of the two is positively correlated (21).

In the process of tilapia farming, it is unknown whether the low oxygen environment affects the infection ability of *L. monocytogenes* to tilapia, and whether the differential expression of *HIF-1α* mediates the infection of *L. monocytogenes* to tilapia by regulating E-cadherin and c-Met, which is the receptor for the InlA and InlB. Therefore, this study focused on assessing the roles of *HIF-1α* in the infection of hypoxic tilapia with *L. monocytogenes*.

## MATERIALS AND METHODS

### The fish and bacterial strains

Tilapia (*Oreochromis niloticus*) juveniles were obtained from the Tilapia Breeding Farm of Guangdong Province (China) and transported to the laboratory. Before experiment initiation, all tested fish were confirmed to be *L. monocytogenes* free following bacteriological examination. Approval was obtained from the Institutional Animal Care and Use Committee of Zhejiang Agriculture and Forestry University prior to using the animals for research. *L. monocytogenes* strains, including the wild-strain EGD-e and gene deletion strains of *InlA* and *InlB* (∆*InlA* and ∆*InlB*), were preserved in our laboratory. All strains were cultivated in Brain Heart Infusion (BHI, Oxoid), shaking at 180 rpm at 37°C.

### Hypoxia exposure experiments and sampling

The healthy tilapia (mean weight = 12.0 ± 3.0 g) were reared in the tanks with a water temperature of 26 ± 0.5°C and dissolved oxygen (DO) concentration at 6.8 ± 0.5 mg/L (the normoxic group) for acclimation of 7 d. Then, a group of tilapia was exposed to tanks constantly aerated with nitrogen gas and oxygen, and the desired DO concentration was set to 2.0 ± 0.5 mg/L (the hypoxic group). In normoxic and hypoxic groups, half of the fish were administered intraperitoneally with 100 µL of EGD-e strain (1.37 × 10^4^ CFU/fish), and the other half were administered intraperitoneally with 100 µL phosphate-buffered saline (PBS) as the control group. The DO level in each tank was measured every 4 h using a dissolved oxygen detector (JPB-70A, China). The nitrite level was <0.5 mg/L, and the pH was 6.5 ± 0.5 in all of the tanks. After hypoxia treatment for 24 h, livers, spleens, and muscles were collected from each group of tilapia and used for detecting *HIF-1α* and its important related gene expression, antioxidant enzyme activity, tissue section observation, and *L. monocytogenes* burden count.

### Liver section observation

After 24-h infection with PBS or EGD-e strain, livers were collected from normoxic and hypoxic fish and cut into a number of 1 mm^3^ slice and fixed in 4% glutaraldehyde for 2 d at 4℃. Fixed samples were routinely dehydrated in a graded acetone series (30–100%), infiltrated with acetone and resin mixture, polymerized in the oven at 60℃ for 48 h, and sectioned. Thin sections were mounted on grids, and post-stained with 2% uranyl acetate for 15 min and washed with distilled water before viewing the samples. The tissue sections were prepared in the Servicebio Biotechnology Co, Ltd. (Wuhan, China) and the samples were viewed using transmission electron microscopy (TEM; HITACHI HT7700, Japan).

### Antioxidant enzyme activity

After 24-h infection with PBS or EGD-e strain, livers were collected from normoxic and hypoxic fish and then ground into pieces with aseptically frosted glass slides in PBS for enzyme activity analysis. Superoxide dismutase (SOD) and catalase (CAT) activities were measured using assay kits (Nanjing Jiancheng Institute, China), according to the manufacturer’s instructions.

### Quantitative real-time PCR

To investigate the changes in *HIF-1α* and downstream gene expression levels, livers, spleens, and muscles were collected from normoxic and hypoxic fish and used to detect *HIF-1α*, *c-Met*, and *E-cadherin* gene expression by qRT-PCR. Total RNA was isolated from livers, spleens, and muscles using the TRIzol reagent (Takara). qRT-PCR amplification with a single dissociation curve peak was performed in a LightCycler 480 Real-Time System (Roche, Switzerland). All the primers are listed in Table 1, and *β-actin* was chosen as an internal standard. The data were analyzed using the 2^−∆∆Ct^ method (22).

**TABLE 1:** Primers used in this study

Name	Nucleotide sequence (5′ to 3′)	Accession number
*HIF-1α*-dsRNA-F	CCCTGTGACCAAGAGGAGC	KY415998
*HIF-1α*-dsRNA-R	GTCATAAACGCGCACGTGAC	
EGFP-dsRNA-F	ATGGTGAGCAAGGGCGAG	U55762
EGFP-dsRNA-R	TTACTTGTACAGCTCGTCC	
*HIF-1α*-F	CTCCAGTCCAGCCAAAGAAG	KY415998
*HIF-1α*-R	AGCTGAAGTCCAGGGAGACA	
E-cadherin-F	CTGCACAGATCGCACAAGAT	MT370500
E-cadherin-R	TGCATTCATGCTCACACTGA	
c-Met-F	GCAGAATTCCCAATCCAGAA	XM005454185
c-Met-R	GCTGTACAACCCCTTGTCGT	
β-actin-F	TCCATTGGCCTTCGTTGC	KC795683
β-actin-R	CTATTCTGTGTGACCCAGG	

### Knockdown of *HIF-1α*

The primers with specific restriction sites for *Hind*III and *Bam*HI were designed based on the known nucleotide sequence of *HIF-1α* (GenBank: KY415998.1). PCR products digested with specific restriction sites were sub-cloned into LITMUS 38i vector (NEB, MA, USA) to gain the recombinant plasmid of L38i-*HIF-1α* and then verified by DNA sequencing. The recombinant plasmid was transformed into HT115 (DE3) cells, and cells were cultured and incubated according to the previous methods (23). After adding IPTG (0.8 mmol/L) to induce expression, bacterial solution with persistent expression of *HIF-1α* protein was subsequently harvested by centrifugation (4,000 rpm, 10 min) at 4°C. The double-stranded RNAs for *HIF-1α* (dsHIF-1α) were purified using a mirVana miRNA Isolation Kit (Ambion, USA). The dsEGPF was used as the negative control. The injection dose of dsHIF-1α (49 µg/fish) was determined by a preliminary experiment. After hypoxia and dsHIF-1α treatment for 24 h, the expression levels of *HIF-1α* in livers, spleens, and muscles were detected by qRT-PCR. If the expression level of *HIF-1α* was knocked down significantly at 24 h post-hypoxia and dsHIF-1α treatment, the mRNA expression levels of *E-cadherin* and *c-Met* were subsequently determined.

### Growth curve of bacterial strains

To evaluate the growth curves, EGD-e, ∆*InlA*, and ∆*InlB* strains were cultured by inoculating into BHI broth and incubated in a shaker bath at 180 rpm at 37°C for 12 h. During the incubation period, the light transmittance of the bacterial solution was measured every 1 h by spectrophotometer, and the growth curves were made according to the OD_600_ value.

### Oxidative stress tolerance assay

To evaluate the antioxidant stress ability of EGD-e, ∆*InlA,* and ∆*InlB* strains, H_2_O_2_ was used as a direct oxidant. EGD-e, ∆*InlA*, and ∆*InlB* strains were cultured overnight in BHI broth. The bacterial suspension was 10-fold serially diluted in PBS, and 10 µL of each dilution was spotted onto BHI agar plates containing various concentrations of H_2_O_2_ (10–15 mM). Following incubation at 37°C for 24 h, colony growth on each plate was assessed and imaged.

### Bacterial adhesion ability *in vitro*

To evaluate the adhesion rate, tissue cells of livers, spleens, and muscles from healthy tilapia cultured in Dulbecco's Modified Eagle Medium containing 20% fetal bovine serum were infected with EGD-e, ∆*InlA,* and ∆*InlB* strains with multiplicity of infection at 10. After 30 min post-infection, tissue cells were washed with tissue culture medium and then isolated by centrifugation. Then cells were serially diluted 10-fold in PBS. A 100 µL sample of each dilution was plated on BHI and incubated at 37°C for over 24 h. The adhesion rates of *L. monocytogenes* to tissue cells were calculated according to the number of colonies.

### Bacterial motility observation

To evaluate the motility ability of bacteria in different dissolved oxygen environments, EGD-e, ∆*InlA*, and ∆*InlB* strains were cultured at 30°C for 24 h under normoxic and anaerobic conditions *in vitro*, respectively. Motility ability was assessed by examining the migration of bacteria through agar from the center toward the periphery of the colony.

### Bacterial burden in organs

To evaluate the bacterial load of EGD-e, ∆*InlA*, and ∆*InlB* under different dissolved oxygen environments, normoxic fish and hypoxic fish were administered intraperitoneally with 100 µL of prepared PBS, EGD-e (1.36 × 10^4^ CFU/fish), ∆*InlA* (1.44 × 10^4^ CFU/fish), or ∆*InlB* (1.39 × 10^4^ CFU/fish), respectively. After 24 h of infection, the livers, spleens, and muscles were collected, homogenized, and serially diluted in PBS. A 100 µL sample of each dilution was plated on BHI and incubated at 37°C for over 24 h. The bacterial count was calculated using the following formula: CUF/g of tissue = numbers of CFUs counted on plate ×dilution factor. To analyze the correlation between bacterial counts and *HIF-1α* expression, the dsHIF-1α treatment hypoxic fish were administered intraperitoneally with 100 µL of PBS, EGD-e (1.40 × 10^4^ CFU/fish), ∆*InlA* (1.30 × 10^4^ CFU/fish), and ∆*InlB* (1.38 × 10^4^ CFU/fish), respectively. The hypoxic fish were administered intraperitoneally with the above strains as the control group. After 24 h of infection with different strains, livers, spleens, and muscles were collected from each group to calculate bacterial count as described above.

### Statistical analysis

In this study, all data are shown as mean ± standard deviation (SD). The statistical analysis was performed using one-way ANOVA followed by Duncan’s test. The comparison of relative gene expression between the two groups was performed using *t*-tests. The differences were considered significant at *P* < 0.05.

## RESULTS

### Effects of hypoxia on the expression of hypoxia-related genes

**Fig 1:**
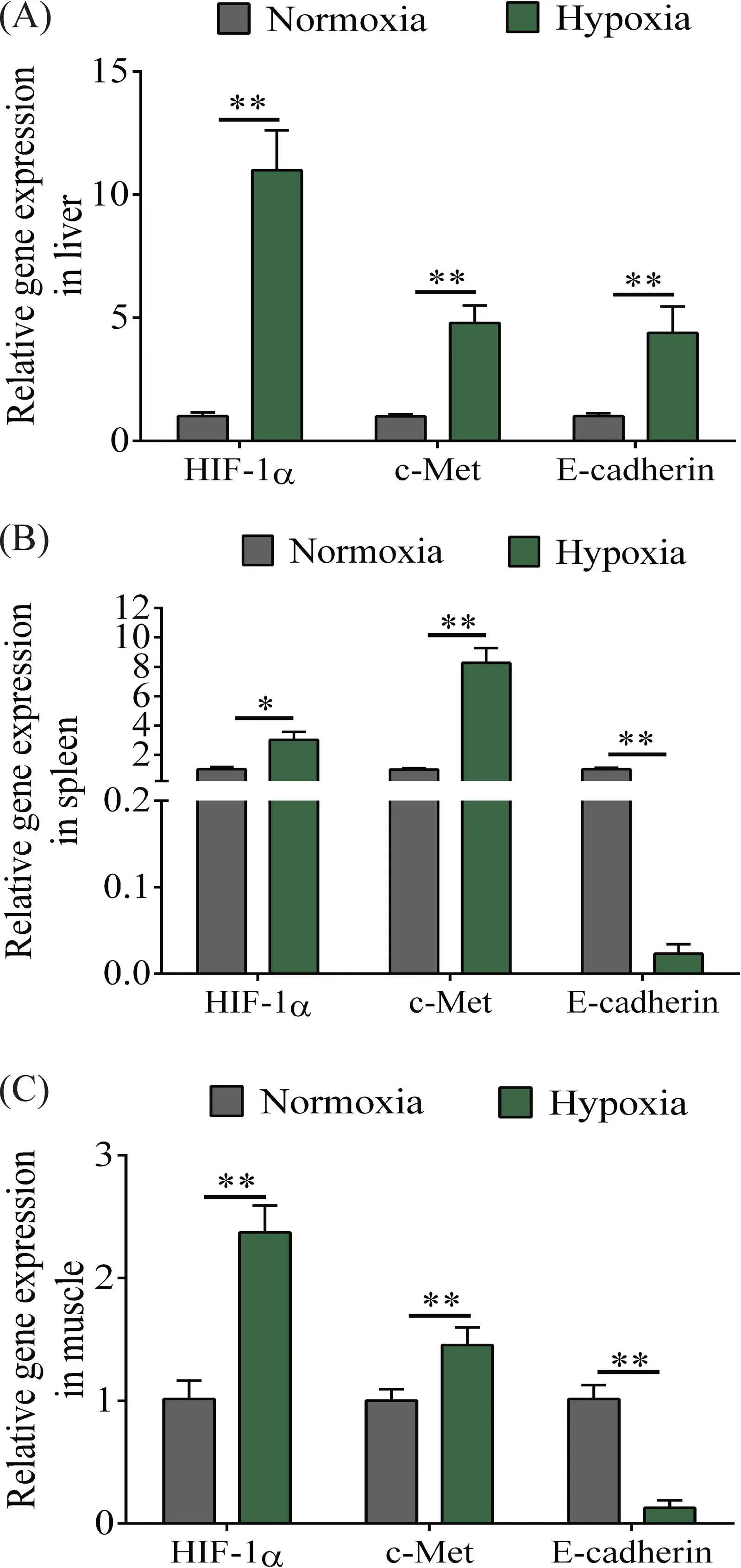
qRT-PCR analysis of *HIF-1α* and receptor-related genes transcription in various tissues of tilapia after hypoxia treatment for 24 h. Data are expressed as mean ± SD (*n* = 3). *P < 0.05; **P < 0.01.

After hypoxia treatment for 24 h, expression profiling of *HIF-1α*, *c-Met*, and *E-cadherin* genes in different tissues of hypoxic fish was conducted by qRT-PCR (Fig. 1). The results showed that the expression levels of *HIF-1α* in all the examined tissues of hypoxic fish were significantly upregulated (*P* < 0.05). It is worth noting that the expression levels of *c-Met* were significantly upregulated in all the examined tissues of hypoxic fish (*P* < 0.05). However, the expression level of *E-cadherin* was only significantly upregulated in the liver of hypoxic fish, and they were significantly downregulated both in the spleen and muscle of hypoxic fish (*P* < 0.05). The significantly heightened expression of *HIF-1α* in important organs suggested that the cellular internal environment was under hypoxic conditions. The significant upregulation of *c-Met* may be correlated with the changes of *HIF-1α* expression level.

### Effects of hypoxia on bacterial count in tissues

**Fig 2:**
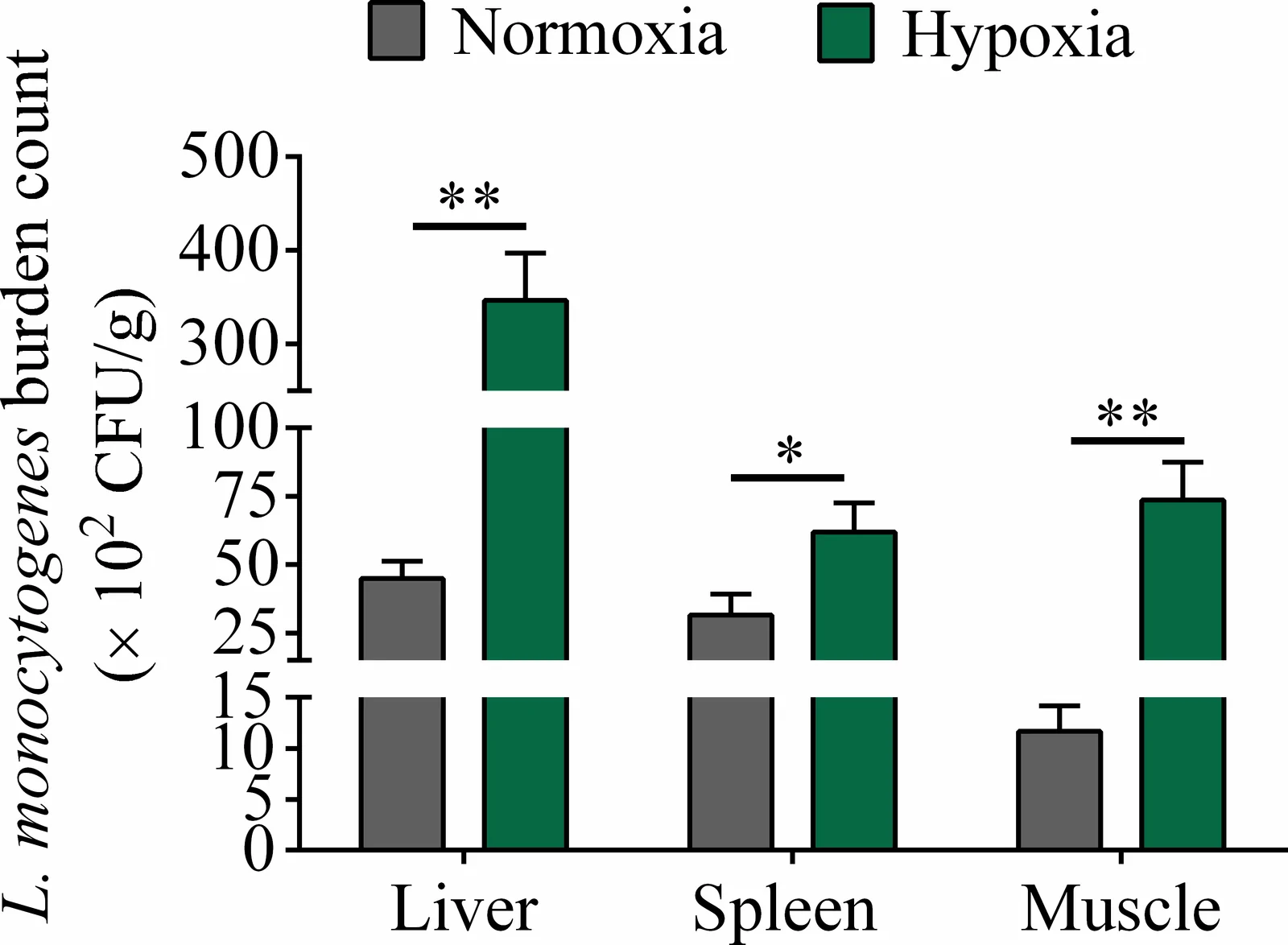
*L. monocytogenes* burden in various tissues of normoxic and hypoxic tilapia. Data are expressed as the mean ± SD (*n* = 3). *P < 0.05; **P < 0.01.

After infection with *L. monocytogenes* of EGD-e strain, tilapia were divided into two groups and reared in normoxic and hypoxic environments, respectively. After hypoxia treatment for 24 h, *L. monocytogenes* were detected in all tissues examined, and the bacterial counts in the livers, spleens, and muscles increased significantly in hypoxic fish compared to normoxic fish (*P* ˂ 0.05; Fig. 2). In addition, the highest bacterial count was in the liver tissue. These findings suggest that tilapia could be infected by *L. monocytogenes* either in normoxic environments or in hypoxic environments, and hypoxic environment may be more conducive to *L. monocytogenes* invasion into tilapia.

### Liver section observation and enzyme activity

**Fig 3:**
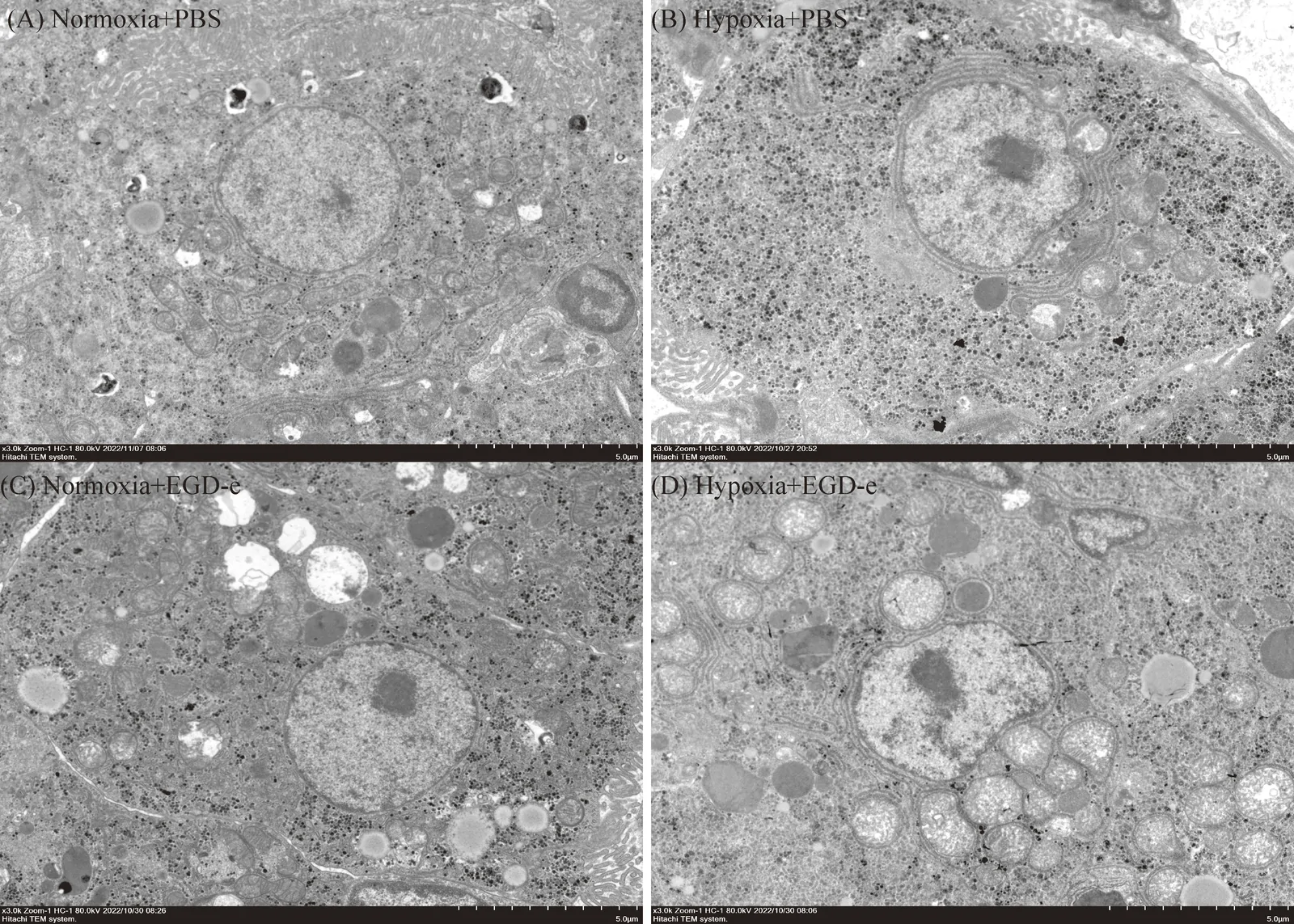
TEM observation of the liver cells from the normoxic and hypoxic tilapia 24 h post-*L. monocytogenes* infection.

**Fig 4:**
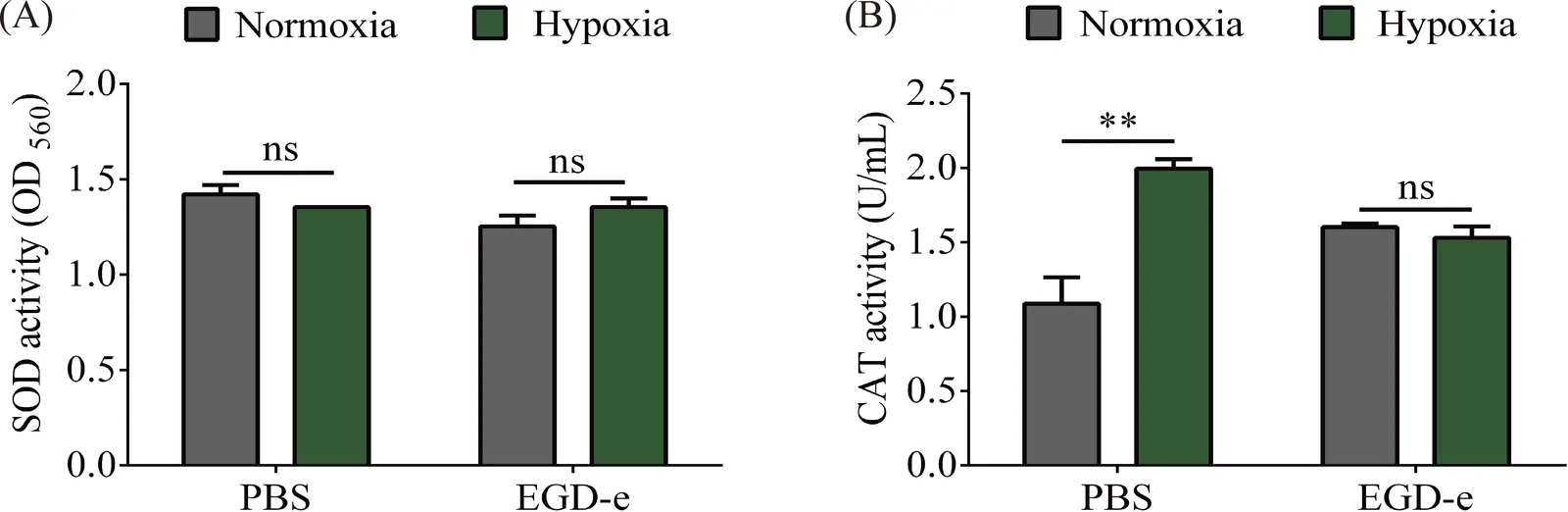
The SOD (**A**) and CAT (**B**) activities in the liver of tilapia after hypoxia treatment for 24 h. Data are expressed as the mean ± SD (*n* = 3). **P < 0.01.

To explore why the bacterial count in the tissues of hypoxic fish increased significantly, we first observed the status of tissue cells by transmission electron microscopy and analyzed the activity of antioxidant enzymes. The tissue section showed that the mitochondria were swollen and the nucleus was atrophic in liver cells of hypoxic fish either infected with EGD-e strain or not. In hypoxic fish infected with EGD-e strain, the number of mitochondria in liver cells increased obviously compared to those treated with PBS (Fig. 3). In the liver of hypoxic fish, CAT activity increased significantly. However, there was no significant difference in CAT and SOD activities between normoxic fish and hypoxic fish infected with EGD-e strain (*P* ˃ 0.05; Fig. 4). These findings suggest that *L. monocytogenes* infection may stimulate mitochondria production and maintain the stable antioxidant enzyme activities under hypoxic conditions. This, in turn, creates a favorable intracellular environment for *L. monocytogenes* infection.

### Effects of *HIF-1α* knockdown on the expression of hypoxia-related genes

**Fig 5:**
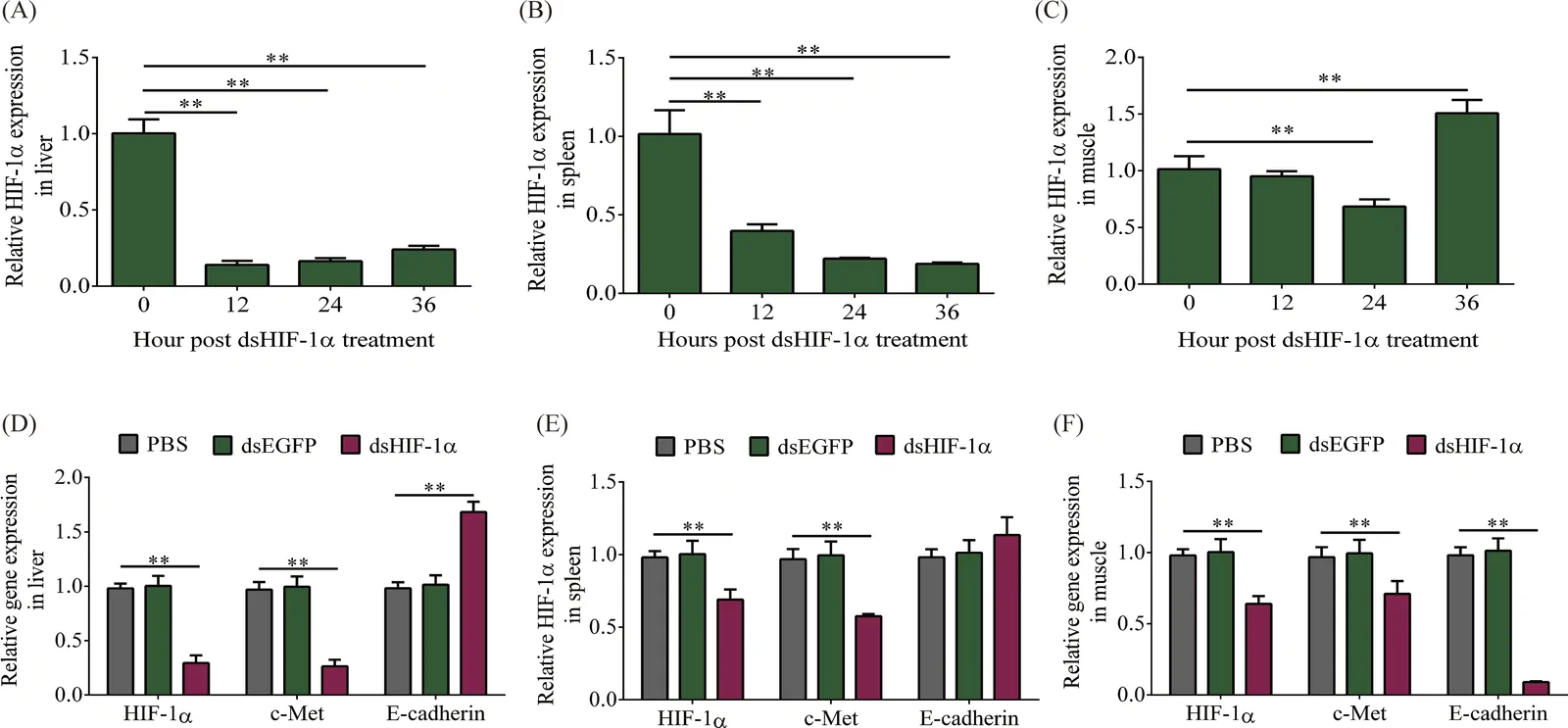
qRT-PCR analysis of *HIF-1α* and receptor-related genes transcription in various tilapia tissues. (**A-C**) *HIF-1α* expression levels after hypoxia and dsHIF-1α treatment at different time points. (**D-F**) The expression levels of *HIF-1α* and receptor-related genes after hypoxia and dsHIF-1α treatment for 24 h. Data are expressed as the mean ± SD (*n* = 3). **P < 0.01.

To further explore whether the expression levels of *c-Met* are regulated by *HIF-1α*, the effect of dsHIF-1α on mRNA expression was determined by qRT-PCR. The effect of dsHIF-1α on *HIF-1α* gene expression in livers, spleen, and muscles at different times post-dsHIF-1α treatment was determined. The results showed that dsHIF-1α inhibited the expression of *HIF-1α* mRNA in livers and spleen from 12 to 36 h post-dsHIF-1α treatment. *HIF-1α* mRNA expressions in all the examined tissues of hypoxic fish were all significantly knocked down at 24 h post-dsHIF-1α treatment (*P* < 0.01; Fig. 5A through C). The relationship between *HIF-1α* expression and the expression of internalin receptor-related genes was also investigated by analyzing the effects of dsHIF-1α on the expression levels of the receptor-related gene in livers, spleen, and muscles of hypoxic fish. The results showed that the expression levels of *c-Met* in all the examined tissues were all significantly downregulated, and the expression level of *E-cadherin* in the liver was significantly upregulated at 24 h post-dsHIF-1α treatment (*P* < 0.05; Fig. 5D through F). These findings suggest that the expression level of the InlB receptor, *c-Met*, may be regulated by *HIF-1α*.

### Biological characteristics of different strains

**Fig 6:**
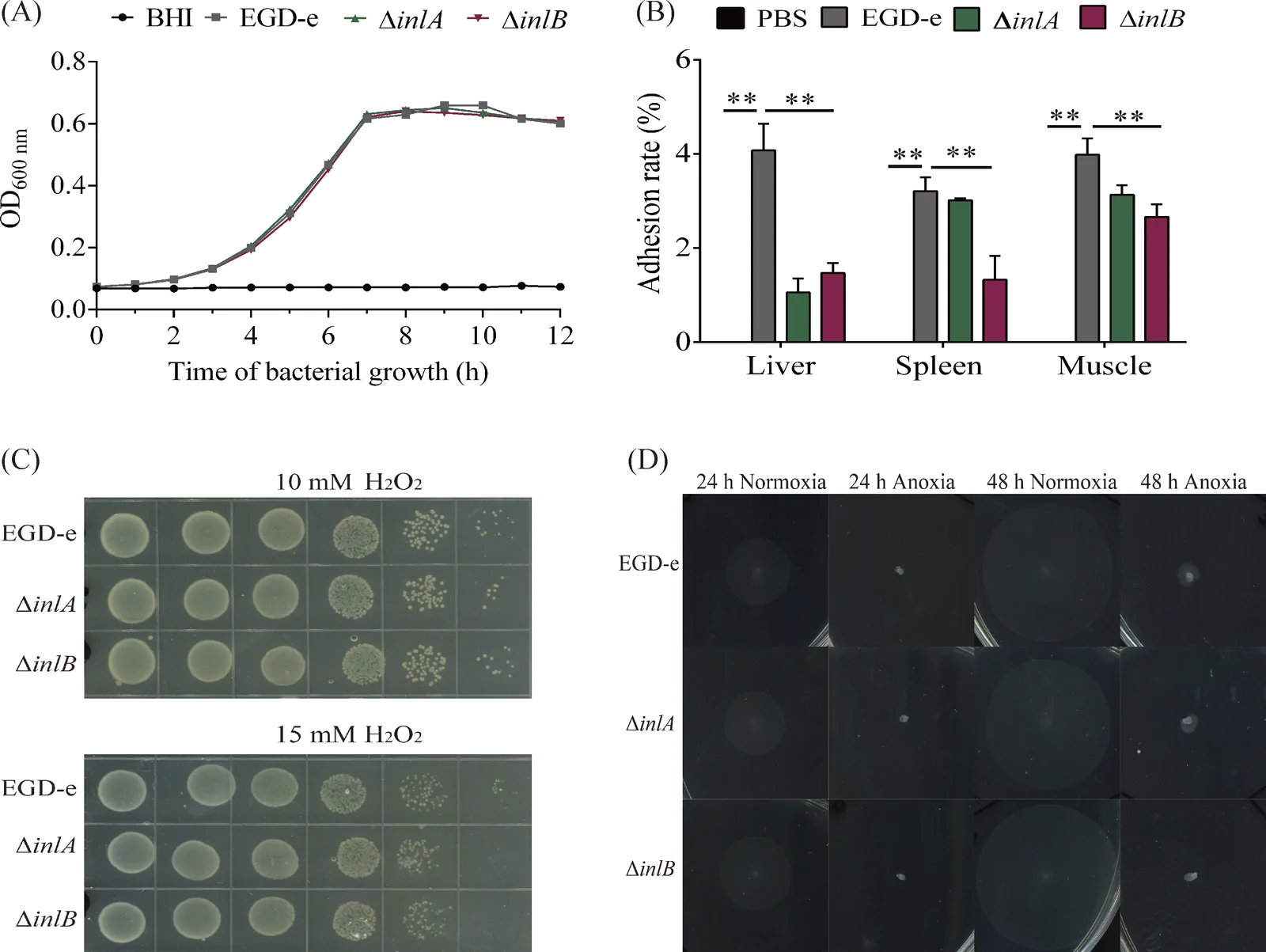
The growth curve (**A**), adhesion rate (**B**), oxidation resistance (**C**), and motility ability (**D**) of EGD-e, ∆*inlA,* and ∆*inlB* strains of *L. monocytogenes*. Data are expressed as the mean ± SD (*n* = 3). **P < 0.01.

E-cadherin and c-Met are the receptors of InlA and InlB, respectively. To explore whether the infection ability of ∆*InlA* and ∆*InlB* to tilapia is affected by hypoxic conditions, biological characteristics of EGD-e, ∆*InlA*, and ∆*InlB* are compared and analyzed. The results showed that there was no obvious difference in the growth curves and antioxidant stress abilities between different strains when the strains were cultured in normoxic conditions (Fig. 6A and C). There was no obvious difference in motility between different strains cultured in anaerobic conditions (Fig. 6D). However, the adhesion rates of ∆*InlB* strain decreased significantly in all the examined tissue cells compared to EGD-e strain (*P* < 0.05; Fig. 6B).

### Effects of hypoxia on the bacterial count of different strains

**Fig 7:**
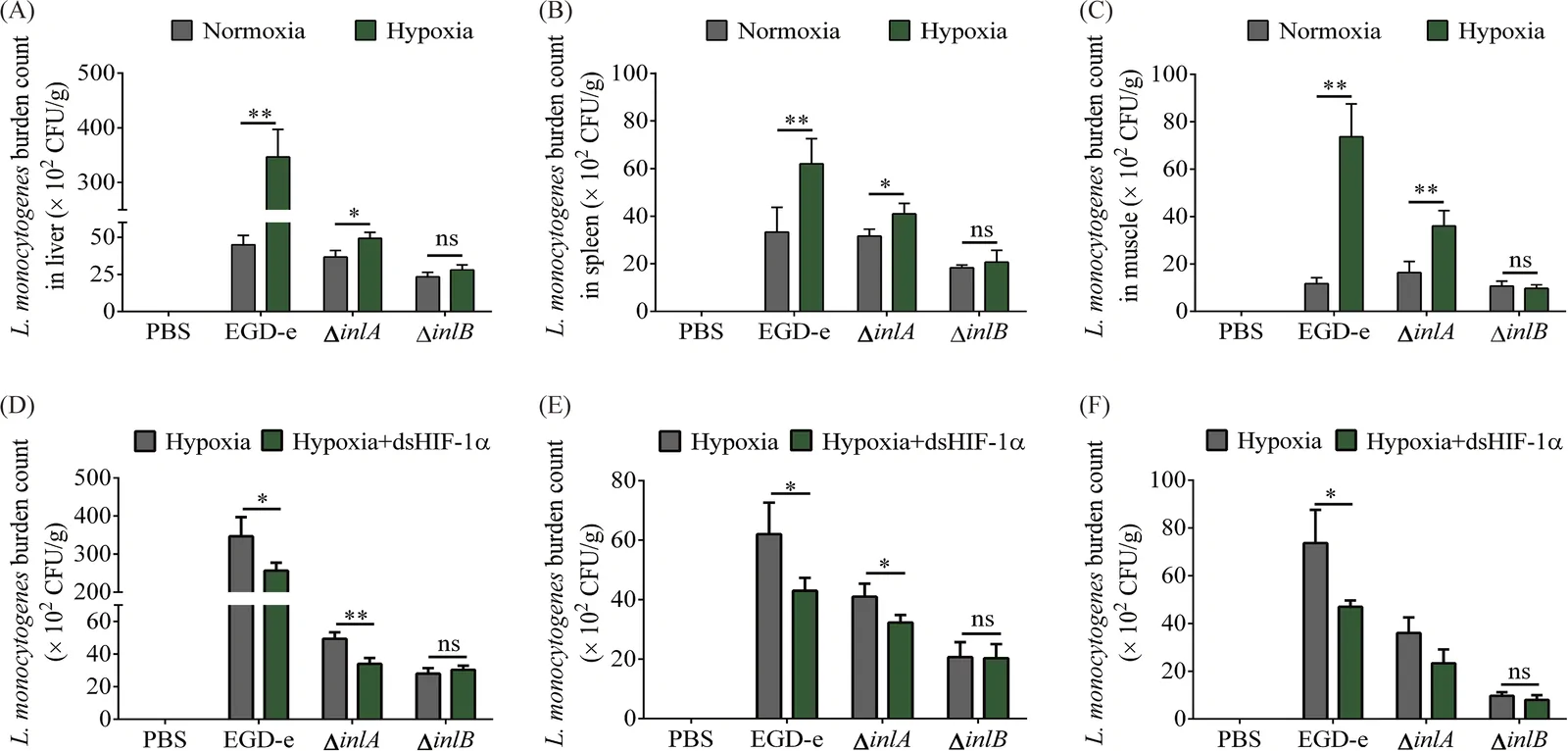
*L. monocytogenes* burden in tilapia various tissues after infection with EGD-e, ∆*inlA,* and ∆*inlB* strains for 24 h. (**A-C**) The bacterial counts in normoxic and hypoxic tilapia. (**D-F**) The bacterial counts in hypoxia and dsHIF-1α treatment tilapia. Data are expressed as the mean ± SD (*n* = 3). *P < 0.05; **P < 0.01.

After infection with EGD-e, ∆*InlA*, and ∆*InlB* strains, tilapia were reared in normoxic and hypoxic conditions. After hypoxia treatment for 24 h, livers, spleens, and muscles were collected to calculate the bacterial count (Fig. 7A through C). The results showed that the bacterial counts in all the examined tissues of hypoxic fish increased significantly compared to normoxic fish after infection with EGD-e and ∆*InlA* strains (*P* < 0.05). However, there was no obvious difference in bacterial counts between normoxic fish and hypoxic fish after infection with ∆*InlB* strain (*P* ˃ 0.05). These results suggested that *L. monocytogenes* burden counts in tissues of tilapia infection with ∆*InlB* strain may not be affected by the hypoxic environments.

### Effects of *HIF-1α* knockdown on the bacterial count of different strains

After 24 h post-treatment with dsHIF-1α, the correlation between bacterial load and *HIF-1α* expression level was analyzed by calculating the bacterial counts in hypoxic fish infected with EGD-e, ∆*InlA*, and ∆*InlB* strains (Fig. 7D through F). The results showed that the bacterial counts decreased significantly in all the examined tissues of hypoxic fish infected with EGD-e and ∆*InlA* strains after dsHIF-1α treatment (*P* < 0.05). However, there was no significant difference in bacterial counts between the PBS group and the dsHIF-1α treatment group in hypoxic fish infected with ∆*InlB* strain (*P* ˃ 0.05). These results suggested that the expression levels of *HIF-1α* in hypoxic fish may affect the invasion ability of *L. monocytogenes* by regulating the expression of the InlB receptor.

## DISCUSSION

The adverse effects of hypoxia on energy metabolism, immune response, and respiratory function have been reported in previous studies (24 – 26). In hypoxic responses, *HIF-1α* activity is tightly regulated by the level of available oxygen in cells (27). The *HIF-1α* transcript level has been identified as a potentially helpful biomarker of aquatic animal exposure to hypoxic conditions (28). As a regulatory factor of nuclear transcription, *HIF-1α* participates in a lot of physiological or pathological processes by regulating the transcription of target genes. Under hypoxic conditions, the increased susceptibility of aquatic animals to pathogenic bacteria may be closely related to the change in *HIF-1α* transcript level (23, 29). To evaluate whether the infection ability of *L. monocytogenes* to tilapia was affected by a hypoxic environment and what was the correlation mechanism between the changes in infection ability and *HIF-1α* transcript level, a series of experiments were carried out involving hypoxic tilapia in the present study.

After hypoxia treatment for 24 h, *HIF-1α* was expressed at significantly high levels in the livers, spleens, and muscles of tilapia, indicating that the tissue cells have been in hypoxic conditions. *L. monocytogenes* burden counts increased significantly in the livers, spleens, and muscles of tilapia under hypoxic conditions, and the highest bacterial counts were in the liver tissue. The liver has a robust metabolic function and plays a vital role in glycogen synthesis and decomposition. The highest bacterial counts in the liver of hypoxic fish may be closely related to the energy supply for bacterial invasion and proliferation by enhanced glycolysis ability of the liver under hypoxic conditions (30). After hypoxia treatment for 24 h, the atrophied nucleus, swollen mitochondria, and increased CAT activity indicated that oxidative stress had occurred in the liver of hypoxic fish (31, 32). After infection of the EGD-e strain, no significant difference in the numbers of mitochondria and CAT activities between normoxic fish and hypoxic fish suggested that *L. monocytogenes* that invade cells may use unique infection mechanisms to maintain the stability of the intracellular environment of hypoxic host for its survival (33).

To further explore the correlation mechanism between significantly increased bacterial counts and the changes in *HIF-1α* transcript level in tilapia under hypoxic conditions, the downstream genes of the *HIF-1α* signaling pathway including *E-cadherin* and *c-Met* were examined by qRT-PCR. E-cadherin is important to maintain tight connections and clearance connections. Overexpression of *HIF-1α* can induce abnormal expression of the *E-cadherin* gene in the oxygen-deprived areas of tumor cells, which leads to the abnormal regulation of the related signaling pathway and affects intercellular adhesion, invasion, metastasis, and survival (16, 18, 29). In this study, whether the abnormal expression of *E-cadherin* promotes the proliferation of bacteria in hypoxic fish by affecting the adhesion between cells remains to be further studied. It is worth noting that the expression levels of *HIF-1α* and *c-Met* were positively correlated in the examined tissues of hypoxic fish. The results are consistent with the previous study that the hypoxia environment in the tumor can promote the upregulation of the *c-Met* expression level (23). The expression levels of *c-Met* were all significantly downregulated when the expression level of *HIF-1α* in the liver, spleen, and muscle of hypoxic fish was successfully inhibited by specific double-stranded RNAs for *HIF-1α* (dsHIF-1α), suggesting that *HIF-1α* might regulate the expression process of *c-Met* under hypoxic conditions. Therefore, we guess that the increased bacterial counts in hypoxic fish may be closely related to the increase in *c-Met* expression levels because the increased *c-Met* expression may significantly improve the binding ability of InlB to the c-Met receptor.

In this study, the ∆*InlA* and ∆*InlB* strains of *L. monocytogenes* were used to further explore the reason for the significant increase in bacterial counts in hypoxic fish. No obvious difference in the growth curves, antioxidant stress, and motility abilities among EGD-e, ∆*InlA*, and ∆*InlB* strains of *L. monocytogenes* suggested that *InlA* and *InlB* deletion do not affect the growth, antioxidant stress, and motility abilities of *L. monocytogenes*, which is a prerequisite for comparing whether the bacterial counts of different strains are affected by the hypoxic environments. After infection of tissue cells with EGD-e, ∆*InlA,* and ∆*InlB* strains, the adhesion rates of ∆*InlB* strains decreased significantly, suggesting that InlB may be the main factor mediating *L. monocytogenes* infection of tilapia. In addition, the bacterial counts of ∆*InlB* strains in examined tissues were not affected by hypoxic conditions and *HIF-1α* expression levels, and the bacterial counts of EGD-e and ∆*InlA* in hypoxic fish decreased significantly when dsHIF-1α inhibited the expression levels of *HIF-1α*. The results are similar to the study of *L. monocytogenes* infection to Hela cells, which showed that the infection ability of *L. monocytogenes* to Hela cells was significantly decreased after inhibition of *HIF-1α* expression (34).

In conclusion, this study sheds light on the relationship between dissolved oxygen levels and *L. monocytogenes* invasion into tilapia, particularly the role of *HIF-1α* in bacterial invasion into hypoxic fish. The findings suggest that *HIF-1α* may promote the internalization of InlB by upregulating *c-Met* expression and result in increased bacterial counts in the livers, spleen, and muscles of hypoxic tilapia. *HIF-1α* plays a key role in promoting the invasion of *L. monocytogenes* into tilapia under hypoxic environments.
